# Embryological Results of Couples Undergoing ICSI-ET Treatments with Males Carrying the Single Nucleotide Polymorphism rs175080 of the *MLH3* Gene

**DOI:** 10.3390/ijms18020314

**Published:** 2017-02-02

**Authors:** George Anifandis, Ourania Markandona, Konstantinos Dafopoulos, Christina Messini, Aspasia Tsezou, Marina Dimitraki, Panagiotis Georgoulias, Alexandros Daponte, Ioannis Messinis

**Affiliations:** 1 Department of Obstetrics and Gynaecology, ART Unit, School of Health Sciences, Faculty of Medicine, University of Thessaly, Larissa 41110, Greece; ranja@hotmail.com (O.M.); kdafop@med.uth.gr (K.D.); pireaschristina@gmail.com (C.M.); dimitrakimarina@yahoo.com (M.D.); dapontea@otenet.gr (A.D.); 2 Department of Molecular Genetics and Cytogenetics, School of Health Sciences, Faculty of Medicine, University of Thessaly, Larissa 41110, Greece; atsezou@med.uth.gr; 3 Department of Nuclear Medicine, School of Health Sciences; Faculty of Medicine, University of Thessaly, Larissa 41110, Greece; pgeorgoul@med.uth.gr

**Keywords:** embryology, embryo quality, pregnancy rates, polymorphism, sperm parameters

## Abstract

Human MLH3 (hMLH3) gene has been suggested to play a role in the DNA mismatch repair mechanism, while it may also be associated with abnormal spermatogenesis and subsequently male infertility. The aim of the present study was to investigate possible relationships between the single nucleotide polymorphism (SNP) rs175080 in the *MLH3* gene of males and the embryological results in couples undergoing intracytoplasmatic sperm injection-embryo transfer (ICSI-ET) treatments. A total of 132 men volunteered for the study and gave written informed consent. All couples were subjected to ICSI-ET treatments in the years 2010 to 2012. The couples were divided into three groups according to the genotype of their husbands: the wild type GG (*n* = 28), the heterozygotic type GA (*n* = 72) and the mutant type AA (*n* = 32). Significantly lower sperm concentration and progressive motility were observed in the AA group as compared to the other two groups (Concentration: 14.57 ± 4.9 mil/mL in AA, 38.3 ± 5.4 mil/mL in GA and 41.03 ± 6.8 mil/mL in GG, *p* < 0.05, mean ± standard error of the mean—SEM). However, significantly better embryological results (mean score of embryo quality–MSEQ) were found in the AA (8.12 ± 0.5) and the GA group (7.36 ± 0.4) as compared to the GG group (5.82 ± 0.7), (*p* < 0.05). Clinical pregnancy rate was significantly higher in the AA genotype group (43.8%) and the GA group (30.6%) than in the GG group (14.3%), (*p* < 0.05). Live birth rate was not different. It is suggested for the first time that the deteriorating effect of the mutant type on sperm characteristics does not impact on embryo development after fertilization in vitro.

## 1. Introduction

Although the causes of infertility can be ascribed to factors from both men and women, mounting clinical evidence indicates that a number of genes and their polymorphisms are implicated in the etiology of infertility in some cases. Specifically, it has been postulated that genes implicated in the mismatch repair (MMR) mechanism may play a role in human fertility [1]. Such genes may also contribute to male infertility through impaired spermatogenesis [2]. Regarding females, it has been suggested that the presence of specific polymorphisms may play a causative role in the unexplained infertility [3]. The proteins of the DNA repair pathway play a crucial role in gametogenesis, while knockout mouse models for the genes involved in repair mechanisms exhibit male and female infertility [1].

Naturally occurring polymorphic variants of both DNA repair genes and the MMR proteins have broadened our knowledge involved in various diseases and genetic defects, including infertility [4,5]. Among the genes that are involved in the process of spermatogenesis, *MLH1* and *MLH3* and their related gene polymorphisms have gained much attention. Their products have been suggested to play directly or indirectly major roles in the process of DNA mismatch repair [6,7]. *MLH1-MLH3*, *MutLγ*, is required for stable crossovers and chiasmata formation and for normal meiotic progression [8]. Despite their central importance in meiosis, much remains to be learned concerning their function and mode of action. In 2000, the human *MLH3* (hMLH3) gene was identified, leading to the production of the *Mut*L homolog protein. This gene was found on 14q24.3 chromosome with a coding length of 4.3 kb [9]. Apart from its involvement in the DNA mismatch repair mechanism, it has been also proposed to play a distinct role in the meiotic recombination mechanism [10,11]. Human *MLH3* gene may be also associated with spermatogenesis and male infertility, as a negative impact of the Pro844 to Leu of the *MLH3* gene (rs175080 polymorphism) on sperm parameters has been recently found [12]. It is likely that various polymorphisms of the MLH3 gene may result in male infertility, based on findings related to the presence of the *MLH3* C2531T polymorphism in infertile men [13]. Moreover, *MLH3-MLH1* pathway seems to play a key role in making crossovers during the process of meiosis [14], since it has been shown that this *MLH3-MLH1* heterodimer is an endonuclease that binds to Holliday junctions [15,16].

Although male infertility can be overcome by the intracytoplasmatic sperm injection (ICSI) procedure, it is well understood that spermatozoa carrying polymorphisms of the genes described above, such as the h*MLH3*, may transfer their polymorphic gene in their offspring. Increased genetic polymorphisms seen in infertile men, raises the potential for these variants to be inherited. Various polymorphisms may confer increased risk of infertility and possible increased risks of somatic defects later in life. Whether the transfer of genetic material has any impact on the fertilization process, the embryo quality, the developmental process or subsequently the in vitro fertilization (IVF) outcome are issues that have to be investigated. 

The aim of the present study was primarily to investigate whether there is any relation of the single nucleotide polymorphism (SNP) rs 175080 in the MLH3 gene of males with the fertilization rate (FR), cleavage rate (CR) and embryo quality. The second end point was to investigate if there is any relation of this polymorphism with pregnancy results (positive hCG, clinical pregnancy rate—CPR and live birth rate—LBR) following embryo transfer (ET) in ICSI cycles. This specific polymorphism of the *MLH3* gene and the possible association with embryological data was investigated for the first time in the Caucasian race. 

## 2. Results

The overall clinical pregnancy rate per cycle was 3033.3% (40/132), while live birth rate per cycle was 18.9% (25/132). Table 1 and Table 2 show the demographic data and the embryological results respectively of the couples studied categorized according to the male genotype. The demographics in terms of age and blood hormonal concentrations (follicle stimulating hormone—FSH, luteneizing hormone—LH, estradiol—E2, prolactin—PRL and testosterone) of men were similar between the three groups. The ages of women were also similar between the three groups. However, the sperm concentration and progressive motility were significantly lower in men with the mutant type AA. While fertilization rate was similar in the three groups, the cleavage rate was significantly higher in the mutant type AA (89.21% ± 4.6%) and the heterozygotic type GA (82.07% ± 3.7%) as compared to the GG genotype (64.89% ± 7.6%, *p* < 0.05). Quality of the embryos, as assessed by the mean score of embryo quality (MSEQ), was higher in the group with the mutant type AA compared to the wild type GG (8.12 ± 0.5 vs. 5.82 ± 0.7, *p* < 0.05) but similar to the heterozygotic type GA (7.36 ± 0.4). 

**Figure 1:**
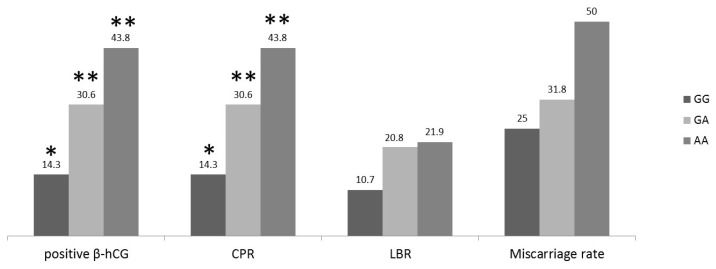
Pregnancy rates per cycle of women (expressed in percentage) according to the genotype of their husbands; The rates of positive β-human chorionic gonadotropin (β-hCG) and clinical pregnancy (CP) of the genotype GG vs. the respective rates of the genotypes GA and AA were significantly lower (*p* < 0.05). Live birth rates (LBRs) were comparable between the three groups (** vs. *, *p* < 0.05).

The pregnancy results are shown in Figure 1. Positive β-human chorionic gonadotropin (β-hCG) and clinical pregnancy rates were significantly higher in women whose the husbands carried the GA or the AA genotypes as compared to women with husbands carrying the GG genotype. The miscarriage rate was 25% (1/4) in the GG genotype group, while in the GA and AA groups 31.8% (7/22) and 50% (7/14) respectively. Live birth rates were comparable between the three groups (Figure 1).

## 3. Discussion

The results of the present study show that the mutant genotype AA of the polymorphism rs175080 in the *MLH3* gene demonstrated relatively better embryological results including the cleavage rate and the embryo quality (which was the first aim), while higher pregnancy rates were observed in the mutation group (the second aim). In this study a total of 132 blood samples of men for this polymorphism were analyzed. In agreement with our previous data, the mutant genotype AA was associated with decreased sperm parameters, including concentration and progressive motility [12]. In contrast to the pregnancy rate, the miscarriage rate was higher in the mutant genotype and as a result the live birth rate was similar with that in the wild type. The present results suggest that the negative impact of the AA genotype on spermatogenesis is not reflected on the morphology and early embryonic development. It is believed that the deteriorating effect of the A allele on spermatogenesis is reversed upon formation of the embryo. 

Although a polymorphism, which was the reversed clone of the present polymorphism of the *MLH3* gene, has been previously studied [14], it was proposed in this study that the mutation in the *MLH3* gene has a negative impact on spermatogenesis and sperm parameters. Our findings confirmed the results of a larger cohort demonstrating the impact of the single nucleotide polymorphism rs175080 of the *MLH3* gene on sperm characteristics [12]. Although the exact mechanism via which the different studied polymorphisms of the gene exert its action on spermatogenesis and sperm parameters is not yet clarified, it is likely that the *MLH3* gene is involved in the maturation process [17]. Moreover, since various genes have been proposed to act as meiosis-involved mismatch repair genes, it is possible that a baseline expression of them is essential for normal male fertility [18]. Abnormal expression of these genes seems to impact negatively on spermatogenesis and sperm parameters [2]. Other authors have also made similar suggestions, since they have shown that polymorphisms of meiosis-involved mismatch repair genes are associated with increased probability of sperm DNA fragmentation and consequently sperm parameters [19]. Recently, it has been indicated that male infertility is associated with at least six polymorphisms, including the rs175080 of the genes that are involved in DNA double-strand break repair and chromosome synapsis [20].

Although it would be expected the presence of sperm DNA mutation to result in impaired fertilization and embryo development, the opposite was found. An explanation for this finding could be the DNA repairing mechanism of the oocytes, which may compensate for the previous mutation. Sperm DNA mutations/polymorphisms that pass to the embryo are very likely to be bypassed by the wild type homologue gene of the oocyte. Similar mechanisms have been suggested in previous studies, between spermatozoa that possess DNA fragmentation and the embryos developed by such spermatozoa [21]. The above suggested mechanism is further validated when comparing the cleavage rate of the groups studied. The mutant type had significantly better cleavage rates (Table 2) and this may be attributed to the fact that after fertilization the embryonic genome is activated. Given the above assumption, it is likely that the wild type of the “maternal” genome eliminates the action of the mutant type of the “paternal” genome and the combined result is the better cleavage rate of the embryos produced, which may explain the better pregnancy rate. Although a higher number of cases are needed it is likely that other genetic or epigenetic factors may determine live birth rate [22]. On the other hand, it is not excluded that the specific *MLH3* polymorphism may have deleterious effects on spermatogenesis during meiosis but positive effects on mitotic divisions during early embryo cleavage. Whether the increased miscarriage rate in the mutant genotype suggests that the potential of the implanted embryo to sustain fetal growth is affected by this “abnormality” needs further investigation. 

Furthermore, one cannot exclude the possibility that the results were affected by the sperm preparation and the ICSI procedures themselves. If sperm preparation could filtrate the spermatozoa carrying the mutation, then this technique may have an important clinical role. In this study, only the ICSI treatment cycles were included. This was done first because of the small number of the IVF cycles in the studied cohort and second in order to overcome possible fertilization failure with the use of IVF. Certainly, a comparison of embryological and pregnancy results between IVF and ICSI treatments in these genotypes might be of importance, however, this should be done in the context of a properly designed randomized trial. Data on the frequency of the studied polymorphism in the women of our cohort were not available therefore the possibility that the heterozygotic state of embryo may be responsible for its competent development cannot be excluded. It might be worth looking at women’s contribution in the context of properly designed trials. Finally, it might be possible that the rs 175080 polymorphism of the *MLH3* gene does not play a major role in early embryonic development.

## 4. Materials and Methods

A total of 132 men of couples that were subjected to 132 consecutive ICSI-ET treatment cycles over a 3-year period (2010 to 2012) were included. This male population was part of a larger group of men studied previously [12]. In the present study, only men of couples that underwent an ICSI-ET treatment cycle were included as male was the main cause of infertility. All patients volunteered for the study and gave written informed consent, while Institutional Review Board approval of the study was also obtained (869/20-02-2009). Ovarian stimulation was achieved by FSH administration in the context of a short or a long gonadotropin-releasing hormone (GnRH) agonist protocol based on women’s individual characteristics.

The 132 men were divided into three groups according to their genotype: the wild type GG (*n* = 28), which was served also as a control group, the heterozygotic type GA (*n* = 72) and the mutant type AA (*n* = 32), while 117 couples finally reached the step of ET. Fertilization or cleavage failure occurred in 15 of them (GG: 6/15, GA: 7/15, AA: 2/15). Genomic DNA was extracted from peripheral blood samples from all men followed by conventional quantitative real time polymerase chain reaction (PCR) for genotyping. 

### 4.1. DNA Extraction

Genomic DNA extraction was performed with the use of the QIAamp DNA Blood Mini Kit (SafeBlood BioAnalytica SA, Athens, Greece). The procedure has been already described previously [12]. 

### 4.2. Genotyping the Single Nucleotide Polymorphism (SNP)

The genotyping of the SNP rs 175080 of the MLH3 gene was performed by using the TaqMan SNP genotyping assay (Applied Biosystems, Life Technologies Corp, Foster City, CA, USA) followed by real-time PCR. The whole procedure was performed according to the protocol recommended by the manufacturer.

### 4.3. Fertilization, Cleavage, Pregnancy Rates and Assessment of Embryo Quality

In order to avoid any bias, all the embryological procedures were performed by two embryologists. Sperm analysis was performed according to WHO, 2010 [23], while sperm preparation was performed according to standard operating procedures and has been previously described [24]. Moreover, ICSI was performed at 16:00 o’clock and the whole procedure has been described [24]. At 16 to 18 h post-ICSI, fertilization rate, expressed as the percentage of oocytes with 2 pronuclei (2PN) to the total number of injected oocytes, was scored. Following cleavage of all normal fertilized oocytes, morphological grade of all embryos was assessed 2 or 3 days post-Ovum Pick Up (OPU). Morphological grade was performed in terms of the number of blastomeres (4–8 blastomeres), grade of fragmentation (in scale 1–4, with 4 representing no fragmentation) and irregularity of blastomeres (in scale 1–2, with 2 representing regular blastomeres) as described previously [24]. For example, one embryo with 8 regular blastomeres and no fragmentation would be assigned a total sum of 14 embryo score, whereas an embryo with 5 irregular blastomeres and 10% to 20% fragmentation (scale 2) would be assigned a total sum of 8 embryo score. For every patient, the cumulative embryo score (CES) was calculated by adding embryo score of each embryo and the mean score of embryo quality (MSEQ) was obtained by dividing the CES with the number of embryos produced. Serum β-hCG levels were measured 12 days post-embryo transfer and a level greater than 15 IU/L was considered positive. Clinical pregnancy was defined when an intrauterine sac was seen by ultrasound 3 to 4 weeks post-hCG. 

The statistical package used was SPSS version 15.0. Data are expressed as mean ± standard error of the mean (SEM). Normality of numeric data distribution has been tested with 1 sample Kolmogorov-Smirnov test. Due to normal distribution, one-way analysis of variance followed by Bonferroni post hoc testing was performed. The frequencies are in Hardy-Weinberg equilibrium. For percentages comparison, chi-square test was performed. An α level of 0.05 was used to determine statistical significance.

## 5. Conclusions

In conclusion, although the sample size is relative small, the present study shows for the first time that, while the polymorphism rs175080 of the MLH3 gene affects negatively the sperm parameters, following fertilization in vitro, the early embryonic development is not deteriorated.

## Figures and Tables

**Table 1:** Baseline measurements from the couples studied according to the genotype of the males.

Variables	Wild Type (GG)	Heterozygotic Type (GA)	Mutant Type (AA)	*p* Value
Number of cycles	28	72	32	
Number of ETs	22	65	30	
Age of men (y)	39.14 ± 1.2	38.5 ± 0.7	37.87 ± 0.9	NS
Age of women (y)	35.89 ± 0.7	34.71 ± 0.5	33.75 ± 0.8	NS
BMI of men (kg/m^2^)	27.76 ± 0.8	27.58 ± 0.4	30.06 ± 0.7	<0.05
Sperm Concentration (10^6^/mL)	41.03 ± 6.8	38.3 ± 5.4	14.57 ± 4.2	<0.05
FSH (IU/L)	7.85 ± 1.4	6.4 ± 0.5	8.3 ± 1	NS
LH (IU/L)	4.73 ± 0.5	4.65 ± 0.2	4.96 ± 0.3	NS
E2 (pg/mL)	45.43 ± 2.8	47.51 ± 1.6	49.23 ± 2.9	NS
PRL (ng/mL)	6.83 ± 0.7	7.39 ± 0.4	8.72 ± 0.9	NS
Testosterone (ng/mL)	4.81 ± 0.3	4.87 ± 0.2	4.53 ± 0.3	NS

BMI: body mass index; FSH: follicle stimulating hormone; LH: luteneizing hormone; E2: estradiol; PRL: prolactin; ET: Embryo Transfer; NS: No significant; y: years.

**Table 2:** Embryological results from the couples studied according to the genotype of the males.

Variables	Wild Type (GG)	Heterozygotic Type (GA)	Mutant Type (AA)	*p* Value
Number of cycles	28	72	32	
Number of ETs	22	65	30	
PRM (%)	39.5 ± 4.1	42.8 ± 2.7	21.06 ± 3.2	<0.05
NPM (%)	12.42 ± 1.9	16.71±1.3	15.75 ± 2.4	NS
IM (%)	43.78 ± 4.4	37.79 ± 2.6	53.81 ± 4.9	<0.05
CoCs	4.7 ± 0.7	5.6 ± 0.4	5.7 ± 0.5	NS
FR (%)	57.64 ± 7.2	61.31 ± 3.3	51.29 ± 4.9	NS
CR (%)	64.89 ± 7.8	82.07 ± 3.7	89.21 ± 4.6	<0.05
CES	17.71 ± 3.3	26.5 ± 2.3	23.43 ± 2.8	NS
MSEQ	5.82 ± 0.7	7.36 ± 0.4	8.12 ± 0.5	<0.05

PRM: progressive motility; NPM: non progressive motility; IM: immotility; CoCs: cumulus oocyte complexs; FR: fertilization rate, CR: cleavage rate, CES: cumulative embryo score, MSEQ: mean score of embryo quality; ET: Embryo Transfer; NS: No significant.
